# Integrated Fermentation, Microbiome and Metabolomic Profiles Reveal Rumen Lipotoxicity Induced by the Masked Mycotoxin Zearalenone-14-Glucoside

**DOI:** 10.3390/toxins18090384

**Published:** 2026-09-06

**Authors:** Zixin Wang, Mingzhu Chen, Jialei Liu, Tao Wang, Zhe Sun, Xiaolu Jin

**Affiliations:** 1 State Key Laboratory of Animal Nutrition and Feeding, College of Animal Science and Technology, China Agricultural University, Beijing 100193, China; wzx717543@163.com (Z.W.); sy20253041094@cau.edu.cn (M.C.); 2 College of Life Sciences, Engineering Research Center of Bioreactor and Pharmaceutical Development, Ministry of Education, Jilin Agricultural University, Changchun 130118, China; shicdh@163.com (J.L.); cagewang@163.com (T.W.); 005242@jlau.edu.cn (Z.S.)

**Keywords:** masked mycotoxins, ZEN-14G, in vitro rumen fermentation, rumen microbiota, lipid metabolism, lipotoxicity

## Abstract

The ubiquitous presence of modified mycotoxins such as zearalenone-14-glucoside (ZEN-14G) in agricultural resources presents a critical challenge to livestock safety, specifically regarding their capacity to disrupt lipid metabolism and fermentation in the rumen. This study aimed to evaluate the dose- and time-dependent direct effects of ZEN-14G on rumen fermentation characteristics, nutrient disappearance, and mycotoxin biotransformation under controlled in vitro conditions, and to explore the associated microbial and metabolic responses. An in vitro batch fermentation system was established with three ZEN-14G doses, namely control (CON), low dosage (GL), and high dosage (GH), across three time points (6, 12, and 24 h). Basic fermentation parameters, fat disappearance rates, and ZEN-14G metabolites were quantified, while 16S rRNA gene sequencing and untargeted metabolomics were conducted specifically on 24 h endpoint samples from the CON and GH groups to investigate downstream mechanistic disruptions. The results showed that while baseline pH homeostasis remained unaffected across groups, ZEN-14G dose-dependently inhibited fat disappearance, with the GH group exhibiting a significant reduction throughout fermentation. Targeted quantification confirmed that ZEN-14G was predominantly deglucosylated to free ZEN and reduced to α/β-ZEL. Endpoint multi-omics revealed that although overall α/β-diversity was maintained at 24 h, ZEN-14G induced fine-scale strain-level replacement (12% shared ASVs), marked by the depletion of the key lipolytic bacterium *Prevotella* sp. R79. Untargeted metabolomics showed marked disruptions in sphingolipid metabolism and linoleic acid oxidation, where the accumulation of cytotoxic oxidized fatty acids (12,13-EpOME) and membrane turnover markers (sphingosine) positively correlated with enriched *Bacillota*. In summary, under macroscopic acid–base homeostasis, masked ZEN-14G directly impairs ruminal lipid metabolism, alters microbial community structure at the strain level, and perturbs cell membrane lipid turnover strictly within an in vitro system. These findings establish a key mechanistic baseline for masked mycotoxin biotransformation in the rumen, highlighting the necessity for future in vivo feeding trials to fully evaluate their systemic risk profile in ruminants.

## 1. Introduction

For a long time, mycotoxin contamination in feed has been a major concern in the livestock industry. Feed contaminated with mycotoxins can lead to a loss of nutritional value and cause animal poisoning, resulting in serious animal health and feed safety issues. At the same time, the presence of toxins and their metabolites in animal products—through residues and transfer along the animal-to-product chain—gives rise to particularly concerning food safety issues, resulting in economic losses for the livestock industry. Consequently, strict testing and control standards have been established to address toxin issues within global agricultural systems. However, cases of poisoning that do not align with conventional monitoring results still occur in the livestock industry. Research has revealed that this phenomenon stems from a class of unique contaminants known as “hidden mycotoxins.” These toxins form covalent bonds with polar molecules such as glucose and sulfate ions, altering their chemical properties and rendering them highly “hidden” in standard detection methods [1]. Zearalenone-14-glucoside (ZEN-14G) is a representative example of such substances [2,3]. Uridine diphosphate-glucosyltransferases (UGTs) in plants catalyze the binding of ZEN to glucose to form ZEN-14G [4]. Unlike the parent compound ZEN, ZEN-14G exhibits significantly increased polarity. These chemical properties make it easily transported and stored within plant tissues. At the same time, due to steric hindrance, ZEN-14G is difficult to detect using conventional toxicity monitoring methods, leading to significant underestimation of its toxicity. Although ZEN-14G is far less toxic than ZEN, studies have shown that it is rapidly hydrolyzed into ZEN in animals [5,6], thereby restoring or even increasing the toxicity. The rumen is a physiological feature unique to ruminants; it serves not only as a highly efficient center for nutrient conversion but also as the first biochemical barrier against the intrusion of foreign substances from feed [7,8]. Concurrently, the complex microbial community in the rumen secretes various digestive enzymes, endowing ruminants with a greater ability to biotransform conventional mycotoxins than monogastric animals [9]. However, this powerful metabolic capacity may not confer an advantage in the metabolism of masked toxins; in the case of masked toxins, the rumen, as the first detoxification organ, may also serve as a site where toxins are activated and potentiated. The rumen fibrous binding microbiota exhibits high expression levels of GH1 and GH3 family β-glucosidase genes [10]. However, β-glucosidase inhibitors can effectively block the deglycosylation and cytotoxicity of ZEN-14G [11]. This suggests that once ZEN-14G enters the rumen, it is likely to be rapidly metabolized by β-glucosidases produced by rumen microorganisms, resulting in the generation of free ZEN. Most existing studies have concentrated on the toxicokinetics and biotransformation pathways of ZEN-14G [12,13,14], while largely neglecting its systemic effects on lipid metabolism, rumen fermentation, and microbial community structure. Notably, ZEN-14G can selectively promote the proliferation of *Bifidobacterium* and *Bacteroides* in the intestinal tract, an effect not observed with ZEN itself [15]. In contrast, other masking mycotoxins have not been found to significantly disrupt the structure of the intestinal microbiota community [16]. These findings suggest that ZEN-14G may exert its biological effects through specific microbe-mediated pathways. However, systematic research on the specific manifestations of this pathway within the rumen, a unique micro-ecosystem, remains insufficient.

Based on this, the present study sought to evaluate the direct metabolic and micro-ecological impacts of ZEN-14G within a simulated rumen environment using an in vitro batch fermentation system coupled with 16S rRNA gene sequencing and untargeted/targeted LC-MS/MS metabolomics. We hypothesized that ZEN-14G acts as a latent precursor undergoing rapid ruminal bioactivation, thereby directly altering local lipid degradation pathways and functional microbial succession without collapsing overall environmental homeostasis. Specifically, this study aimed to evaluate: the dose- and time-dependent biotransformation kinetics of ZEN-14G into parent ZEN and its phase I reduced metabolites; the direct effects of ZEN-14G exposure on fundamental fermentation parameters, nutrient disappearance, and fatty acid oxidation; and the targeted shifts in microbial community structure and membrane lipid metabolic profiles under controlled in vitro conditions. By establishing these empirical in vitro baselines, this work provides a rigorous mechanistic foundation for re-evaluating the biological risks of masked mycotoxins in ruminant nutrition.

## 2. Results

### 2.1. ZEN-14G Primarily Inhibits Lipid Loss in a Dose-Dependent Manner

**Figure 1:**
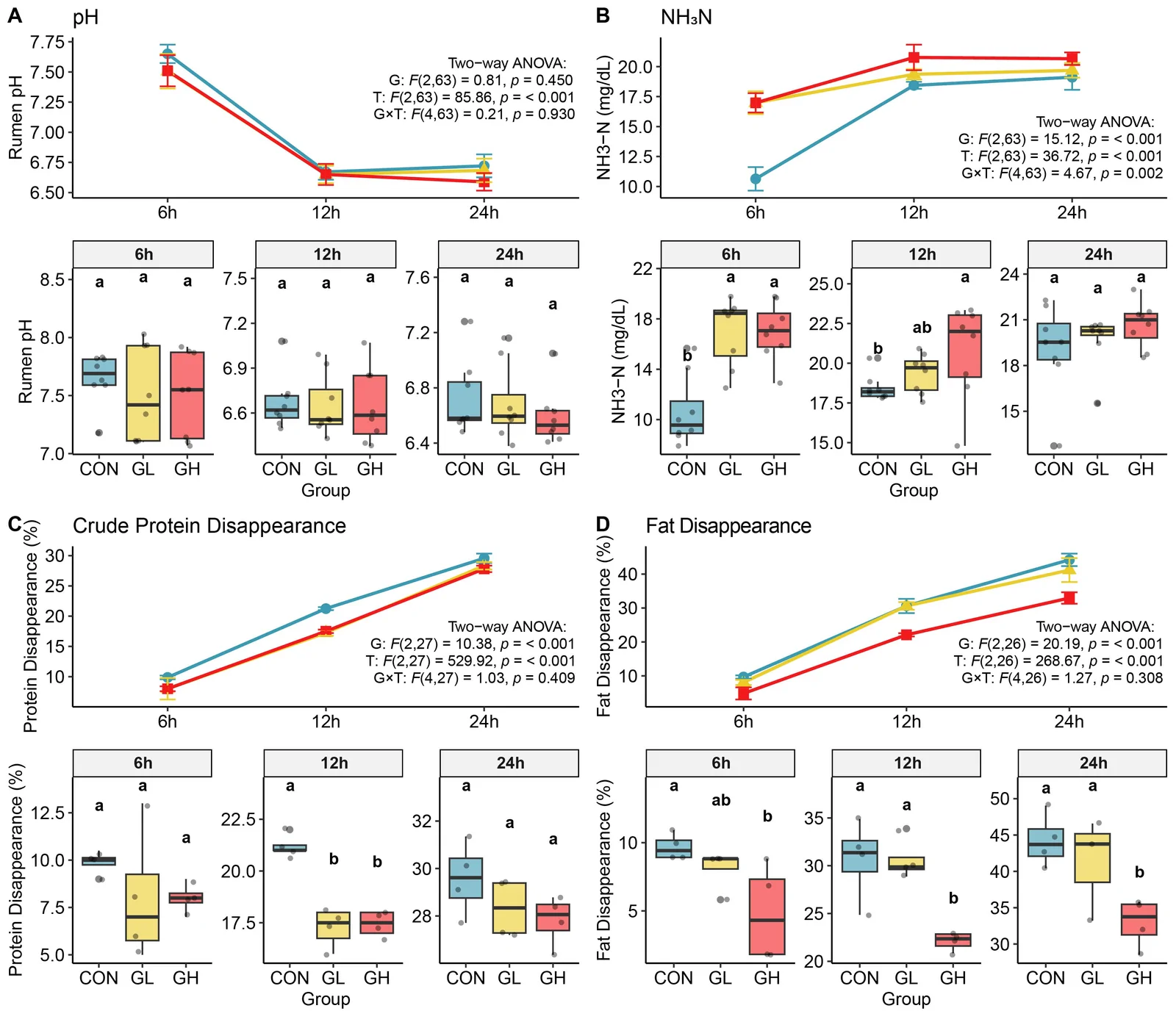
Changes in rumen fermentation parameters and nutrient digestibility rates were assessed after 6, 12, and 24 h of in vitro rumen digestion of zearalenone-14-glucoside (ZEN-14G) at varying concentrations (0, 1.5, 15 mg/kg). (**A**) Rumen pH, (**B**) ammonia nitrogen (NH_3_-N), (**C**) crude protein disappearance, and (**D**) crude fat disappearance were analyzed. Each parameter is illustrated using a composite graph: the trend line at the top depicts the dynamic changes in each parameter over fermentation time; italicized text within the graph denotes the *F*-values and *p*-values for the treatment effect (G), time effect (T), and their interaction (G × T) derived from the two-way ANOVA. The lower box-and-whisker plots display the distribution of values across different treatment groups at each time point; the scatter points represent biological replicates, and values marked with different lowercase letters (e.g., a, b, ab) indicate statistically significant differences among groups (Fisher’s LSD test, *p* < 0.05), whereas those sharing common letters denote non-significant differences. The legend at the bottom applies to all subfigures, where CON (blue), ZEN-14G low-dose group (GL, 1.5 mg/kg, yellow), and ZEN-14G high-dose group (GH, 15 mg/kg, red) denote the control group, low-dose group, and high-dose group, respectively.

During the fermentation process, various parameters in rumen fluid—including pH, NH3-N, crude protein and crude fat digestibility, and ADF and NDF digestibility—collectively determine rumen fermentation efficiency, internal environmental homeostasis, and ultimate production performance. This study measured various parameters in three treatment groups within an in vitro fermentation system at multiple fermentation time points (6, 12, and 24 h). During fermentation, pH values in all groups showed a significant downward trend over time (*p* < 0.05), but there were no significant differences between groups, indicating that the ZEN-14G treatment did not disrupt the acid–base balance of the fermentation system (Figure 1A). Ammonia nitrogen content was significantly affected by both toxin treatment and time (*p* < 0.001). The ammonia nitrogen content in the 6 h (GL, GH) treatment groups was significantly higher than that in the CON group, indicating that the toxin addition caused a transient shock to the rumen microbial system, leading to impaired microbial nitrogen assimilation (i.e., reduced utilization of ammonia for microbial protein synthesis) in the short term; however, by 24 h, the ammonia nitrogen content in all groups tended to converge, suggesting that the rumen microbiota gradually adapted and recovered its nitrogen utilization capacity with fermentation time (Figure 1B). Protein digestibility was affected by both toxin treatment and fermentation time, with no interaction between the two (Figure 1C). Two-way ANOVA indicated that fat disappearance was significantly influenced by both dose and time (*p* < 0.001) (Figure 1D). Compared with the control (CON) group, the high-dose (GH) treatment group significantly suppressed fat loss rates throughout the entire fermentation cycle (6, 12, and 24 h) (*p* < 0.05), suggesting potential impairment of production performance. This highlights the strong impact of ZEN-14G toxicity on lipids, leading to the hypothesis that lipids are the primary target of ZEN-14G toxicity in the rumen. ADF digestibility was significantly affected by fermentation time (*p* < 0.001). Comparisons of intergroup differences at fixed time points revealed that at 6 h, ADF digestibility in the CON and GH groups was higher than that in the GL group, suggesting that a low-dose ZEN-14G environment may influence ruminal fiber metabolism (Figure S1A). Meanwhile, NDF digestibility increased across all groups over time, with no significant differences between groups. This suggests that although ZEN-14G may transiently affect fiber metabolism in the early stages of fermentation, the microbial system possesses a recovery capacity, and no adverse effects on overall fiber digestibility are observed at 24 h (Figure S1B).

In vitro fermentation results indicate that ZEN-14G selectively modulates the in vitro rumen ecosystem, with this selective regulation primarily focused on rumen lipid metabolism. Contrary to the conventional understanding that masked toxins primarily harm rumen microorganisms, these findings provide strong evidence that, in actual production settings, ZEN-14G exerts its toxicity in the rumen primarily through specific damage to lipid degradation. This has significant implications for the health of ruminants and the nutritional quality of their by-products.

### 2.2. High-Dose ZEN-14G Alters the Rumen Fluid Microbiota Within 24 h

**Figure 2:**
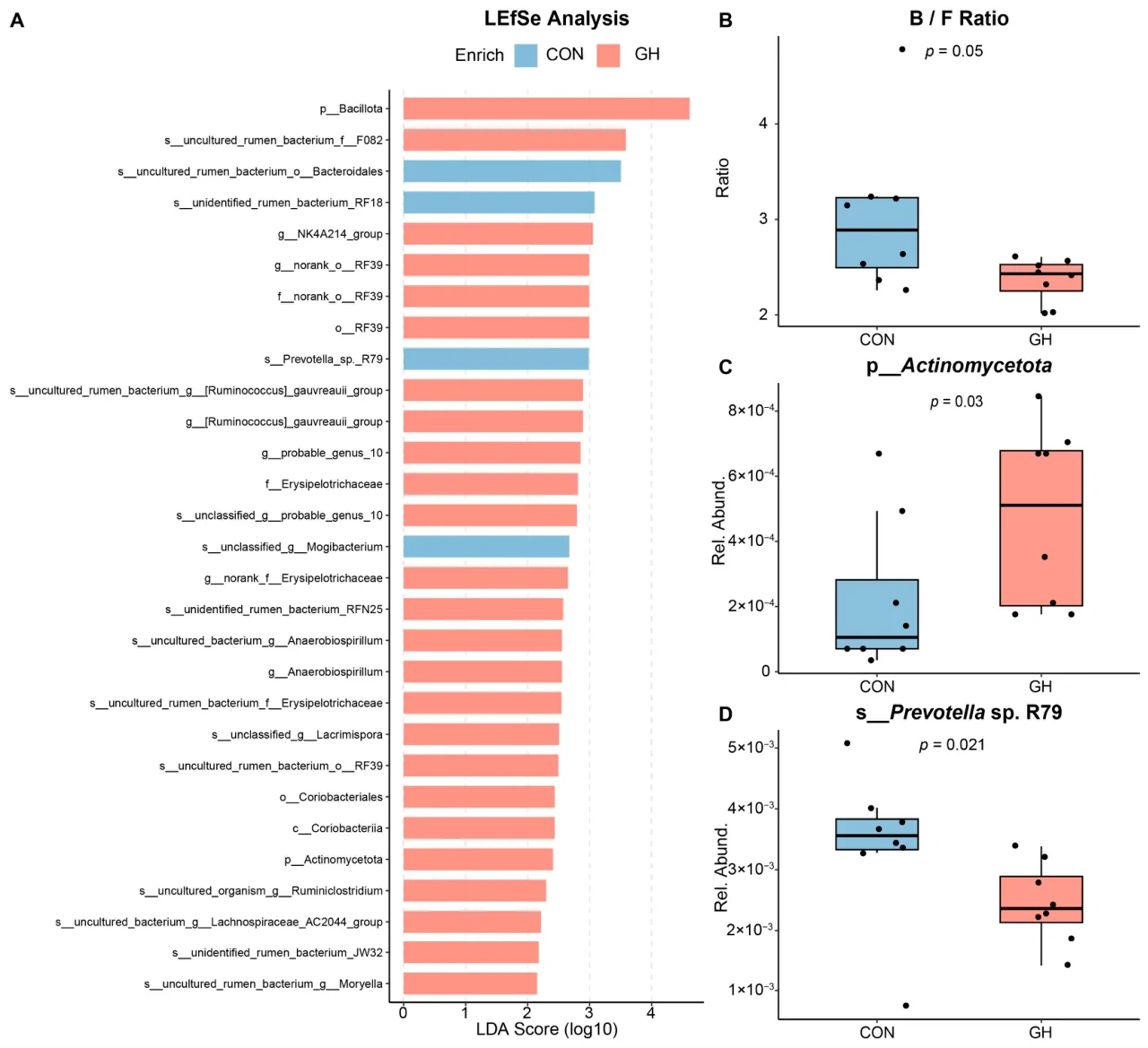
Specific effects of a 24 h treatment with ZEN-14G (0 and 15 mg/kg) on the key microbial taxa and ratios in the rumen microbial community. (**A**) LEfSe analysis. This analysis identifies bacterial taxa with significant differences from the phylum to the species level (LDA > 2.0, *p* < 0.05). (**B**) B/F ratio analysis. This analysis presents the relative abundance ratio of the *Bacteroidota* to the *Bacillota* phyla (Wilcoxon test). (**C**) *Actinomycetota* abundance. This analysis shows changes in the relative abundance of the *Actinomycetota* phylum, assessed using the Wilcoxon rank-sum test. (**D**) *Prevotella* sp. R79 abundance. This analysis reveals changes in the relative abundance of *Prevotella* sp. R79. Statistical significance for differential microbial relative abundance (e.g., *Prevotella* sp. R79) was evaluated using the non-parametric Wilcoxon rank-sum test, whereas comparisons for normally distributed continuous variables across box plots were assessed using independent-samples Student’s *t*-tests.

To further investigate the effects of ZEN-14G on the rumen microbial community, we also performed 16S sequencing. The results for α-diversity (Figure S2A) showed that there were no statistically significant differences (*p* > 0.05) in α-diversity indices (including the Ace, Chao1, Shannon, and Simpson indices) between the CON and GH groups after 24 h of fermentation. This indicates that the ZEN-14G treatment did not compromise the overall richness and diversity of the original microorganisms in the fermentation environment. Venn diagram analysis revealed the fine-tuned regulation of fermentation broth microbial community composition by the ZEN-14G treatment at different taxonomic levels (Figure S2B). At the ASV level, the overlap between the CON and GH groups was only 12%, indicating that the toxin primarily regulates the microbial community through strain-level replacement or specialization; at the species level, the number of shared species between the two groups increased to 292, accounting for 61.5%, indicating that although there was significant variation at the strain level, most of this variation was concentrated within certain shared species; that is, the toxin did not significantly affect the core composition of the two treatment groups at the species level. At the lowest resolution, the genus level, the community structures of the two treatment groups converged further, with shared genera accounting for 70.3% of the total, far exceeding the shared proportion at the ASV level. A comprehensive analysis indicates that differences in the microbial communities between the CON and GH groups are primarily reflected at the intraspecific or closely related species (ASV) level. This suggests that toxins primarily regulate microbial communities through strain-level replacement or specialization, whereas at higher taxonomic levels such as species and genus, the two groups maintained a high degree of core group conservation, indicating that toxins have a limited impact on the macroscopic bacterial classification of the fermentation broth. The results of the PERMANOVA analysis of microbial β-diversity (Figure S2C) showed no significant differences in microbial community composition between the CON and GH groups, indicating that the toxin had no significant effect on the dominant core microbial communities in the rumen. The results of the LEfSe multi-level species-level discrimination analysis based on 16S sequencing (LDA > 2.0, *p* < 0.05) (Figure 2A) showed that among the microbial taxa exhibiting significant differences between the CON and GH groups, *Prevotella* sp. R79 (*Prevotella* sp.), which was significantly enriched in the CON group, was significantly suppressed in the GH group (Figure 2D). The decline in its abundance directly impaired fat metabolism efficiency, indicating that exposure to ZEN-14G caused significant disruption to the rumen microbiota structure of the fermentation broth, and the induced dysbiosis pattern exhibited high specificity; in contrast, compared to the CON group, the GH group showed significant enrichment of *Bacillota*. It is speculated that its efficient conversion of carbohydrates has optimized energy acquisition pathways, indirectly leading to a decrease in fat conversion rates in a fermentation environment. Concurrently, *Actinomycetota* was also significantly enriched in the GH group (Figure 2C), indicating a community shift toward fiber degradation and secondary metabolism; this also suggests that under high-dose ZEN-14G conditions, the decrease in *Prevotella* sp. R79 and the increase in *Actinomycetota* jointly inhibit lipid metabolism in the fermentation broth. It is also speculated that the decrease in the *Bacteroidota*/*Bacillota* (B/F) ratio (Figure 2B) is caused by ZEN-14G inducing a shift in the focus of microbial metabolism from fat/protein to a fiber-utilization pattern dominated by the *Bacillota* phylum, manifesting as a metabolic preference for “fiber over lipids.” This indicates that metabolic activity of rumen microorganisms under ZEN-14G exposure shifts toward efficient carbohydrate utilization. From the perspective of phylum-level composition (Figure S3A), *Bacteroidota* and *Bacillota* dominate in both groups, indicating that high ZEN-14G supplementation did not disrupt the basic microbial ecosystem structure of the rumen. However, combined with the results of LEfSe analysis, it can be concluded that the high-ZEN-14G treatment altered the microscale dynamics of rumen lipid metabolism by upregulating *Bacillota*/*Actinomycetota* and downregulating *Prevotella* sp. R79, thereby reshaping the microscale dynamics of lipid metabolism in the rumen microbiota (Figure S3B).

The above results confirm that, although treatment with high doses of ZEN-14G did not disrupt the original microbial abundance and diversity in the fermentation environment, nor did it have a significant effect on the dominant and core microbial communities in the fermentation broth, it did significantly affect the abundance of various microorganisms at the strain level—including *Bacillota*, *Actinomycetota*, and *Prevotella* sp. R79—thereby reducing the efficiency of lipid metabolism in the entire system.

### 2.3. Metabolomic Profiling Reveals High-Dose ZEN-14G-Induced Alterations in Rumen Lipid Metabolism

**Figure 3:**
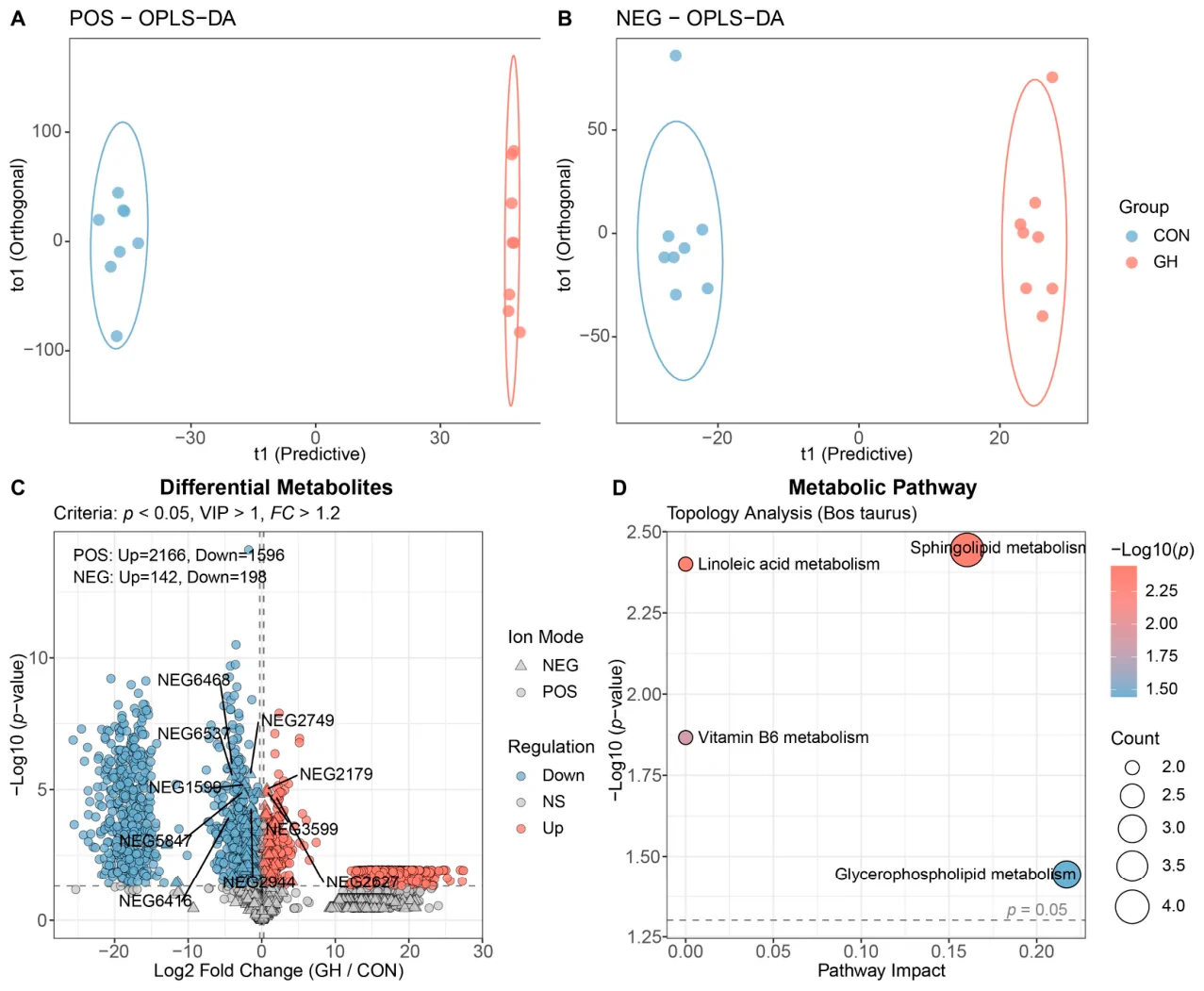
Alterations in the rumen metabolome profile induced by ZEN-14G treatment. (**A**,**B**) Orthogonal Partial Least Squares Discriminant Analysis (OPLS-DA) score plots of (**A**) positive-ion mode (POS) and (**B**) negative-ion mode (NEG). The t1 axis represents predictive variation between groups, and the to1 axis represents orthogonal variation. (**C**) Volcano plot of differential metabolites (POS and NEG combined). Red dots indicate significantly upregulated metabolites, and blue dots indicate downregulated metabolites (criteria: *p* < 0.05, VIP > 1, *FC* > 1.2). The top 10 metabolites ranked by VIP scores are labeled. (**D**) Metabolic Pathway Topology Analysis. The bubble plot displays the enriched KEGG pathways (library: Bos taurus). The x-axis represents the pathway impact score, and the y-axis represents the significance level (−log10 *p*-value). Bubble size indicates the number of hit metabolites, and color intensity indicates statistical significance.

The PCoA results show that, in both positive- and negative-ion modes, the sample points from the GH group and the CON group are clearly separated (Figure S4A,B), indicating that high-dose ZEN-14G treatment significantly altered the metabolic profile of ruminal fermentation; simultaneously, the OPLS-DA results demonstrated that the between-group differences were far greater than the within-group individual variations (Figure 3A,B), confirming the reliability of the rumen metabolomics data. Meanwhile, we identified a large number of differentially abundant metabolites (Figure 3C). The results showed that in the positive-ion mode, 2166 metabolites were upregulated and 1596 were downregulated, while in the negative-ion mode, 142 were upregulated and 198 were downregulated.

KEGG pathway enrichment analysis of differentially expressed metabolites revealed (Figure 3D) that the metabolites differing between the GH and CON groups were primarily concentrated in pathways related to lipid metabolism. Among these, linoleic acid metabolism, sphingolipid metabolism, and glycerophospholipid metabolism were significantly enriched; in particular, the linoleic acid metabolism pathway was highly enriched, suggesting that it plays a central regulatory role in the response to high-dose ZEN-14G treatment.

### 2.4. Correlation Analysis Between Metabolomics and 16S

**Figure 4:**
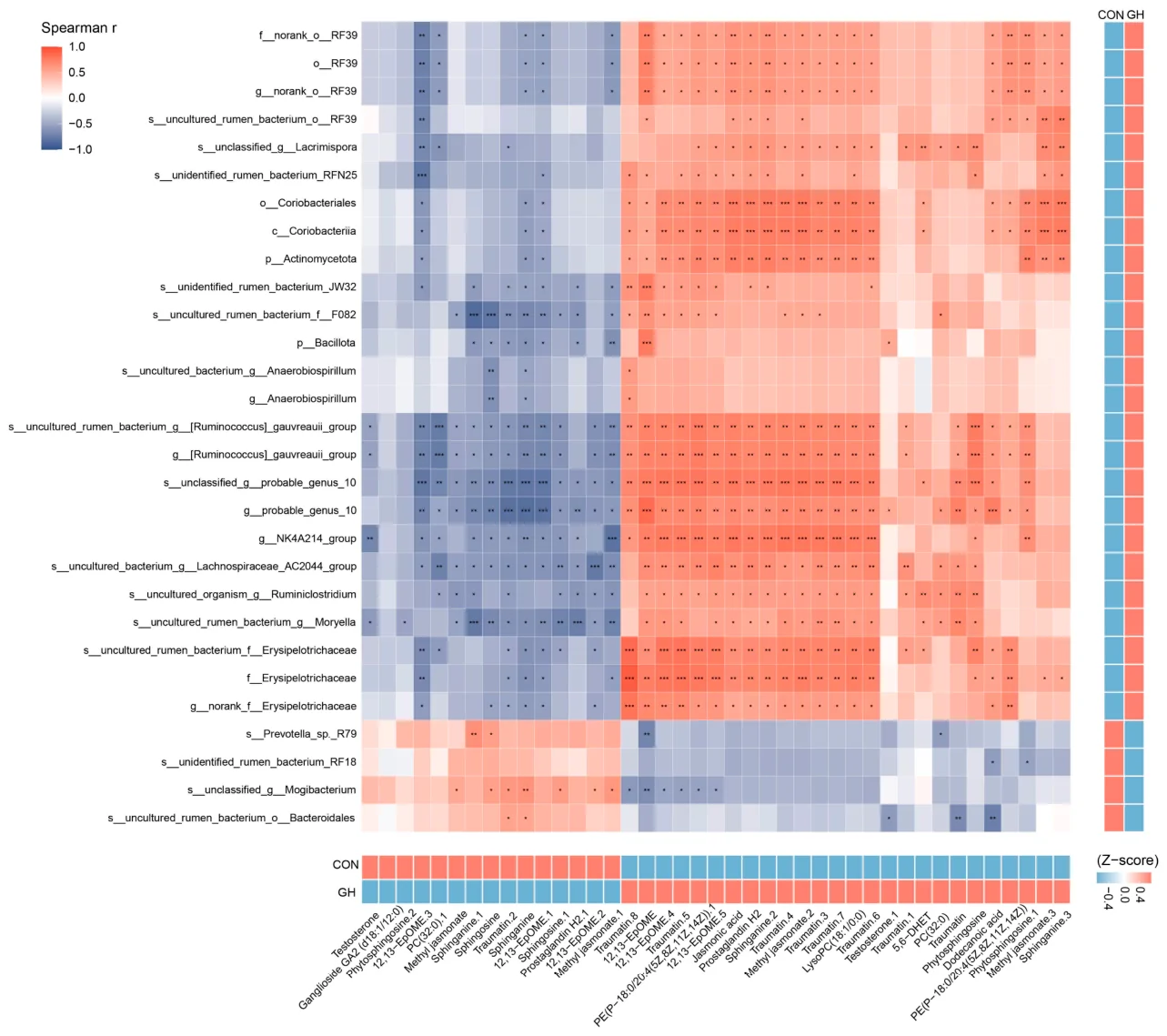
Correlation analysis between differentially abundant bacterial taxa and lipid-related metabolites in vitro, conducted 24 h after treatment with ZEN-14G (0 and 15 mg/kg). The heatmap illustrates the Spearman rank correlation coefficients between differentially abundant bacterial taxa (rows, spanning from phylum to species levels) identified by LEfSe and lipid metabolites (columns) annotated by KEGG. The selection criteria included an LDA greater than 2.0 for bacterial taxa and a VIP exceeding 1 for metabolites, which are also required to be involved in KEGG lipid metabolism pathways. The color legend indicates the strength and direction of the correlation: red signifies a positive correlation, while blue denotes a negative correlation; asterisks indicate statistical significance (* *p* < 0.05, ** *p* < 0.01, *** *p* < 0.001).

The results of the Spearman correlation analysis (Figure 4) indicate that there is an association between the differentially abundant microbial taxa and metabolites in fluid between the GH group and the CON group. The heatmap reveals a clear dichotomy: *Bacillota*/*Actinomycetota*, which was significantly enriched in the GH group, showed a highly significant positive correlation with major lipid metabolites, whereas *Prevotella* sp. R79, which was significantly enriched in the control (CON) group, generally exhibited a negative correlation with these metabolites (Figure 4).

### 2.5. Major In Vitro Ruminal Metabolites of ZEN-14G

**Figure 5:**
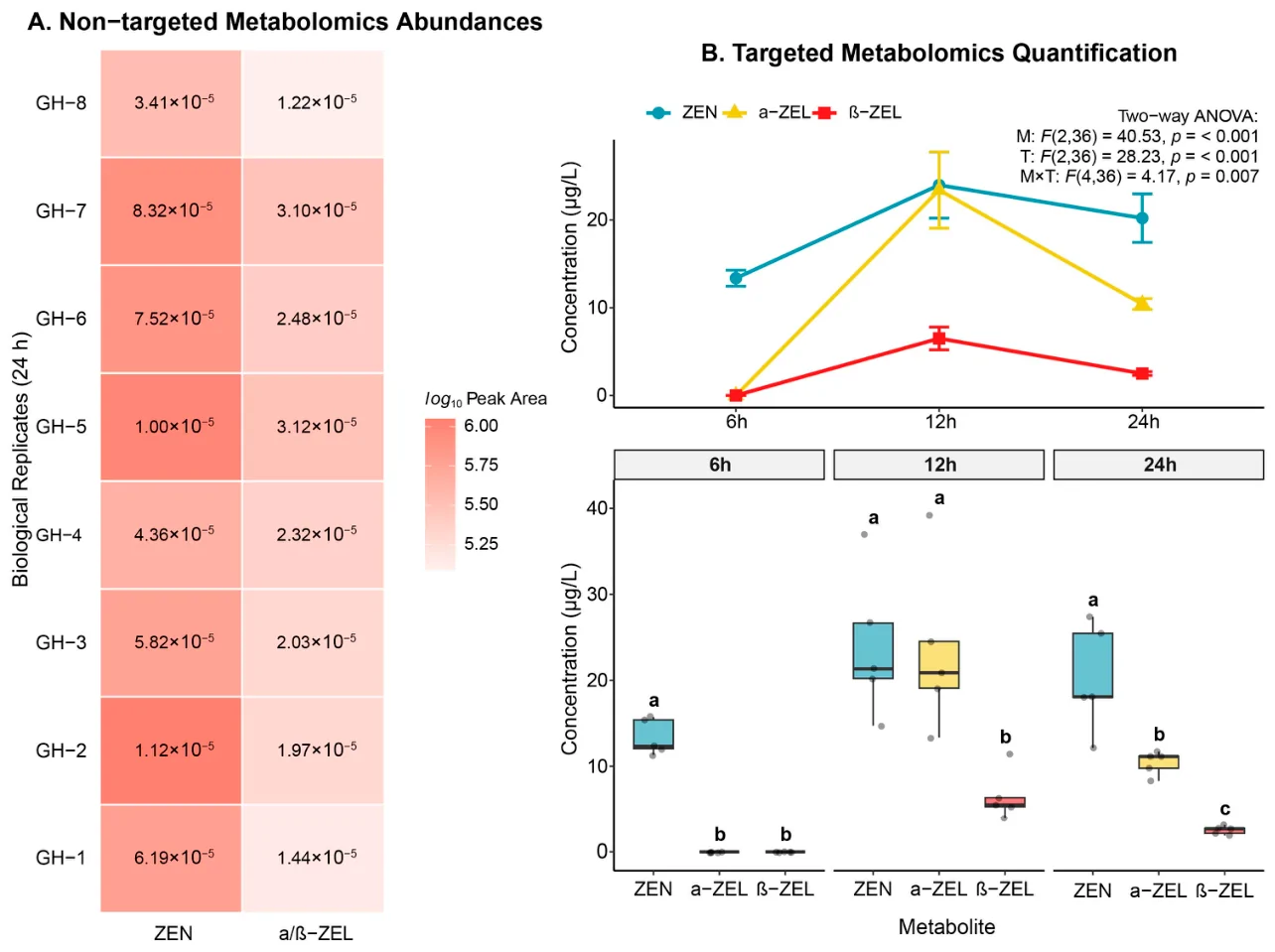
In vitro metabolic transformation and absolute quantification of ZEN-14G in the rumen fermentation system. (**A**) Non-targeted metabolomics abundances: A tile heatmap showing the raw peak areas (log10-transformed for visualization) of ZEN and a/b-ZEL across eight biological replicates after 24 h of fermentation. The text inside the cells displays the raw peak area values formatted in scientific notation. (**B**) Targeted metabolomics quantification: Composite profiles illustrating the absolute concentration of ZEN, a-ZEL, and b-ZEL over the fermentation cycle. The top line graph depicts the dynamic transformation of the three target compounds, with italicized annotations representing the two-way ANOVA results. The bottom boxplots show the concentration distribution of each compound at 6, 12, and 24 h. Jittered dots represent individual biological replicates (*n* = 5). Different lowercase letters (a, b, c) above the boxes indicate significant differences (Fisher’s LSD, *p* < 0.05).

To elucidate the metabolic patterns of ZEN-14G in the rumen microbiome, this study first employed non-targeted metabolomics in conjunction with a custom-built PCDL (containing 27 Phase I/II metabolites of ZEN-14G) [17]. A full-spectrum scan was performed on the fermentation broth (Figure 5A). Under the current chromatographic conditions and within the 27-compound coverage of our self-constructed PCDL suspect library, no other complex biotransformation products or adduct metabolites were identified beyond ZEN and its primary reduction products (α/β-ZEL). The results showed that, in the in vitro culture system, ZEN-14G exhibited highly directed metabolic conversion, with the major metabolites preliminarily identified as ZEN, α-ZEL, and β-ZEL; no other more complex or adduct metabolites were detected. Subsequently, targeted metabolomics was used to perform absolute quantification of the aforementioned candidate metabolites (Figure 5B), which confirmed that ZEN-14G undergoes rapid de-glycosylation under the action of rumen microorganisms. The core metabolic pathway in the in vitro fermentation system primarily involves conversion to free ZEN, accompanied by the generation of small amounts of hydrogenation reduction products, namely α-ZEL and β-ZEL, with α-ZEL being the predominant product.

## 3. Discussion

### 3.1. ZEN-14G Is a Potent Inhibitor of Ruminal Lipid Metabolism

In the early stage of fermentation (6 h), the NH3-N content in the GL and GH groups was significantly higher than that in the CON group, indicating a transient toxic shock to the rumen microflora. Exposure to high concentrations of ZEN-14G temporarily impaired the nitrogen assimilation capacity of rumen bacteria, preventing them from efficiently utilizing ammonia nitrogen for microbial protein synthesis. This led to the short-term accumulation of free NH3-N in the fermentation broth, rather than the acceleration of protein degradation. However, with the extension of fermentation time to 24 h, the NH3-N levels in all groups converged. This trend indicates that the rumen microbial community possesses high functional resilience and adaptability; through population dynamics adjustments and metabolic acclimation, the microflora successfully adapted to the toxic environment and restored their nitrogen assimilation efficiency, leading to the recovery and convergence of NH3-N levels (Figure 1B).

### 3.2. The Relationship Between Microbial Changes and Lipid Metabolic Disorders

We propose that the decrease in the *Bacteroidota*/*Bacillota* (B/F) ratio reflects a compensatory niche shift in the rumen microbial community in response to ZEN-14G stress. Under normal circumstances, *Bacteroidota* plays a major role in the degradation of lipids and proteins [18], while *Bacillota* is highly efficient at converting carbohydrates [19]. The precise toxicity of ZEN-14G selectively suppressed *Bacteroidota* (specifically lipid-utilizing groups like *Prevotella* [20]), thereby hindering lipid loss. To compensate for this energy barrier, the rumen microbial ecosystem underwent a compensatory niche adjustment, promoting the enrichment of *Bacillota* and *Actinomycetota*. This shift prioritized carbohydrate degradation and secondary fiber utilization, optimizing alternative energy acquisition pathways to maintain overall metabolic homeostasis. This explains why total VFA levels remained stable despite the severe impairment of fat digestibility under ZEN-14G exposure, representing a transition from a lipid-degrading state to a highly carbohydrate-utilizing state (Figure 2).

### 3.3. Disorders from Cell Membranes to Signaling Molecules

Sphingosine is a key intermediate in sphingolipid metabolism. Metabolomic analysis results (Figure 3D) show that sphingolipid metabolism was significantly enriched in the GH group, suggesting a potential key pathway responding to ZEN-14G treatment in the in vitro fermentation system. This finding is highly consistent with the result showing only a 12% overlap in ASV levels between groups (Figure 1B), suggesting that ZEN-14G induced large-scale strain-level replacement of the microbial community within the system; as a core component of bacterial cell membranes, the high enrichment of sphingolipids in the GH group is directly linked to the disintegration and turnover of biofilms during the strain-level replacement process [21]. The Spearman correlation analysis results (Figure 4) show that key metabolites such as sphingosine are significantly positively correlated with *Bacillota*/*Actinomycetota* members highly enriched in the GH group. This transition from structural to signaling components holds profound biological significance. The release of sphingolipids not only marks the remodeling of microbial community structure but also points to their possible role as endogenous signaling molecules mediating the rumen microbiota’s response to ZEN-14G. The metabolic pathway shifts resulting from the turnover of these structure-specific components provide a novel perspective for understanding how masked toxins exert their toxic effects by influencing microbial ecological structure and, consequently, altering metabolic products.

### 3.4. The Metabolic Fate of Masked Toxins in In Vitro Fermentation Systems

In this study, the abundance of *Prevotella* sp. R79 in the GH group was significantly lower than that in the CON group. We propose that this selective suppression, as seen in our in vitro fermentation system, is driven by a “suicide-activation” feedback loop. During the early stages of fermentation, *Prevotella* sp. R79 actively secretes highly active β-glucosidases to hydrolyze the glycosidic bond of ZEN-14G [22], aiming to utilize the glucose moiety. However, this hydrolysis process de-glycosylates the masked toxin, releasing free ZEN and its active derivative α-ZEL [23,24]. The localized accumulation of these highly toxic free estrogens exerts strong toxic feedback that selectively suppresses *Prevotella* sp. R79 itself. Thus, the active de-glycosylation of ZEN-14G by *Prevotella* sp. R79 leads to suppression of its own population, representing a classic “suicide-activation” feedback inhibition mechanism (Figure 5).

### 3.5. Comprehensive Toxicological Pathways of the Hidden Form of Zearalenone

In summary, this study utilized an in vitro fermentation system to model a ZEN-14G induced comprehensive toxicity pathway that may occur in the rumen. It should be noted that in the present screening, no higher-order biotransformation products or novel conjugated adducts were characterized beyond free ZEN and α/β-ZEL. While this finding underscores hydrolytic deglucosylation and reduction as the dominant metabolic fates of ZEN-14G in the rumen ecosystem, this observation must be interpreted within the methodological boundaries of our study. Specifically, our self-curated PCDL suspect library was built upon 27 known Phase I and Phase II derivatives. Therefore, the non-detection of other complex conjugates does not entirely preclude the trace existence of uncharacterized microbial metabolites or novel matrix adducts that fall outside the library’s spectral coverage or below the instrumental detection sensitivity. Future studies integrating untargeted high-resolution feature mining, isotopic labeling, and deeper non-targeted MS/MS fragmentation will help capture any potential trace biotransformation intermediates. Unlike the conventional understanding that mycotoxins exert their toxicity by causing collapse of the microbial community, the deleterious effects of ZEN-14G are highly selective and subtle. This toxic pathway stems from precise strain-level replacement; by inhibiting key lipolytic bacteria, ZEN-14G disrupts the initial stage of lipid turnover without altering overall diversity. In this study, the conversion of ZEN-14G into free ZEN and its reduced Phase I metabolites (α-ZEL and β-ZEL) is consistent with the emerging theory of “latent mycotoxin bioactivation” in ruminants. Although glycosylation significantly reduced the direct estrogenic activity of ZEN-14G, rumen microbial β-glucosidases hydrolyzed the glycosidic bond, releasing free ZEN into the fermentation substrate. Importantly, the subsequent microbial reduction of ZEN to α-ZEL is a critical toxicological bioactivation step, as α-ZEL exhibits several-fold higher binding affinity for bovine estrogen receptors than the parent compound ZEN. Although the formation of β-ZEL is generally regarded as a low-affinity detoxification bypass, the coexistence of these two reduction pathways suggests that the rumen microbiome not only degrades latent toxins but also actively regenerates and enhances their toxicological and endocrine-disrupting hazards. These findings provide a basis for understanding the potential link between the biotransformation kinetics of latent mycotoxins and functional toxicological risks in ruminant nutrition. This triggers a cascade of metabolic reactions: impaired lipid hydrolysis leads to the abnormal accumulation of 12,13-EpOME—a metabolite of linoleic acid oxidation—in the environment, while intense bacterial turnover induces massive cell membrane turnover, releasing highly concentrated sphingolipid signaling molecules. Ultimately, these disrupted metabolic products, combined with the dysregulated microbial structure, collectively constitute a functionally impaired rumen microenvironment. This specific disruption of lipid metabolism not only explains the persistently declining fat disappearance rate but also raises the potential threat that long-term intake of ZEN-14G may pose to energy utilization efficiency and milk and meat quality in ruminants. This study not only combines microbiology and metabolomics to elucidate the specific mechanism by which ZEN-14G inhibits the function of an in vitro fermentation system, but also offers a perspective that calls into question traditional toxin safety evaluation theories by revealing strain-level precise replacement under macroscopic homeostasis and the resulting metabolic shifts. The identification of this comprehensive toxicological pathway not only provides a new perspective and basis for the scientific assessment of the biosafety of hidden mycotoxins but also offers a critical theoretical foundation for the future development of novel animal health protection strategies aimed at stabilizing ruminal metabolic function, holding significant implications for ensuring the healthy production of modern ruminants.

### 3.6. Study Limitations and Future Perspectives

It should be acknowledged that this study has several limitations. First, to maximize the sensitivity of the analysis and elucidate the downstream mechanisms of ZEN-14G induced lipotoxicity, 16S rRNA sequencing and untargeted metabolomics analyses were prioritized for the 24 h endpoint samples to compare the CON group and the GH group. The GL group was excluded from the multi-omics screening because preliminary fermentation experiments indicated that significant inhibition of lipid degradation and phenotypic disruption occurred primarily in the GH group; therefore, ultra-high-dose analysis was adopted as a standard and cost-effective screening protocol. However, this design failed to characterize the microbiome and metabolome across dose-dependent gradients and early-time dynamics. Moreover, the use of an in vitro batch culture system represents a methodological consideration that should be taken into account when contextualizing our results. This model does not fully reproduce the physiological complexity of the intact rumen–host axis, particularly regarding ruminal outflow and absorption, epithelial barrier function, hepatic biotransformation, renal excretion, and systemic immune responses [25]. Taken together, our findings provide meaningful mechanistic information regarding the direct microbial and metabolic perturbations elicited by ZEN-14G in the ruminal environment. Given the inherent differences between in vitro and in vivo systems, these results are best interpreted as in vitro-specific phenotypic responses rather than direct evidence of systemic effects in live ruminants. Therefore, caution is warranted when extrapolating these microbial-level findings to live animals. Future in vivo feeding trials covering multiple dose gradients, sequential time points, and host–microbiome interactions are necessary to comprehensively validate the systemic biosafety of masked mycotoxins.

## 4. Conclusions

This in vitro study provides evidence that ZEN-14G does not exert its toxicity by disrupting the overall ruminal microenvironment (e.g., pH, fiber digestibility), but rather acts as a highly selective inhibitor of lipid metabolism, precisely targeting lipid-degrading bacterial communities. High doses of ZEN-14G significantly reduced the abundance of *Prevotella* sp. R79, a key lipid-degrading bacterium in the fermentation broth, resulting in a sustained decrease in fat removal efficiency. While microbial community structure remained stable at the genus level, large-scale strain-level replacement occurred at the ASV level, with compensatory enrichment of the *Bacillota* and *Actinomycetota* phyla, leading to the accumulation of linoleic acid oxidation products (such as 12,13-EpOME) and abnormal activation of the sphingolipid metabolic pathway. This cascade effect, ranging from strain replacement to metabolite remodeling, suggests a unique toxic pathway of ZEN-14G: by inhibiting specific functional strains, it triggers the turnover of cell membrane components and the release of lipid signaling molecules, ultimately impairing the rumen’s lipid turnover capacity. This study offers a perspective that challenges the conventional understanding of mycotoxins as primarily broad-spectrum antimicrobial agents. It raises the hypothesis that cryptic toxins could influence energy metabolism in ruminants through “precision targeting” rather than “global disruption,” providing a critical theoretical basis for re-evaluating the biosafety of cryptic mycotoxins and developing intervention strategies aimed at stabilizing rumen function. However, given the absence of host physiological processes (e.g., absorption, hepatic clearance, and systemic immunity) in this experimental system, these mechanistic insights should be considered hypothesis-generating for in vivo scenarios and require careful validation through future animal feeding trials before any systemic extrapolation can be made.

In summary, this study provides solid in vitro experimental evidence for the specific metabolic fate and microbiological effects of ZEM-14G in a simulated rumen ecosystem. Under closed batch fermentation conditions, ZEN-14G undergoes complete microbial deglycosylation and downstream reduction into free ZEN and α/β-ZEL. High doses of ZEN-14G directly inhibit rumen fat loss and interfere with fatty acid oxidation without disrupting the overall acid–base balance. Rather than causing generalized microbial collapse, ZEN-14G induces precise strain-level replacement (depleting the key lipolytic strain *Prevotella* sp. R79) accompanied by compensatory enrichment of *Bacillota* and *Actinomycetota*, which directly correlates with the accumulation of cytotoxic oxidized fatty acids (12,13-EpOME) and sphingolipid membrane turnover markers. These findings demonstrate that within an in vitro system, masked ZEN-14G impairs ruminal lipid metabolism through targeted functional bacterial suppression and membrane lipid remodeling. Crucially, these mechanistic insights strictly reflect the direct microbial and biochemical responses of the in vitro model; future in vivo feeding trials incorporating epithelial absorption, hepatic clearance, and systemic immune interactions are required before broad physiological extrapolations can be drawn.

## 5. Materials and Methods

### 5.1. Rumen Fluid Sources

Rumen fluid was collected from five Simmental bulls in good health, of similar weight, and aged 2–3 years immediately after slaughter at a local commercial abattoir. Because the samples were obtained as by-products of standard commercial meat production, specific animal ethics committee approval was not required for this study. Prior to slaughter, the animals underwent a 14-day pre-feeding period at the Jilin Agricultural University to stabilize the rumen microbial community. To simulate the rumen environment of lactating dairy cows, the diet was a total mixed ration (TMR) formulated according to the Chinese “Dairy Cow Feeding Standard (GB/T 32755-2016),” satisfying the nutritional requirements of a 550 kg cow producing 20 kg of milk daily. Animals were fed twice daily at 08:00 and 18:00 with ad libitum access to water. The basal diet was confirmed to be free of mycotoxins. Detailed raw material composition and nutritional profiles of the diet have been described in our previous study [26].

### 5.2. Chemicals

All solvents and reagents were of analytical or chromatographic grade. Acetonitrile (ACN, catalog No. A955-4) and methanol (MeOH, catalog No. A456-4) were obtained from Fisher Chemical (Thermo Fisher Scientific, Waltham, MA, USA). ZEN1-4G (purity ≥ 99%, catalog No. BePure-26554), ZEN (purity ≥ 99%, catalog No. BePure-26548), α-ZEL (100 μg/mL in acetonitrile, catalog No. BePure-26544XA), and β-ZEL (100 μg/mL in acetonitrile, catalog No. BePure-26546XA) were purchased from BePure (Beijing, China).

### 5.3. In Vitro Rumen Fermentation

#### 5.3.1. Experimental Design

This experiment utilized the in vitro batch culture method to simulate rumen fermentation. Three treatment groups were established: (1) control (CON) group: only the toxic solvent methanol was added; (2) low-dose latent toxin (GL) group: 1.5 mg/kg (dry matter basis) of ZEN-14G was added; (3) high-dose concealed toxin (GH) group: 15 mg/kg (dry matter basis) of ZEN-14G was added. Each group consisted of 8 repetitions.

#### 5.3.2. Preparation of Fermentation Buffer

The rumen fermentation buffer was prepared following the established method of Menke and Steingass and was divided into three components [27]: Solution A, Solution B, and Solution C.

Solution A: Prepare one day in advance. The components include K_2_HPO_4_ (382.50 mg/L), KH_2_PO_4_ (292.00 mg/L), (NH_4_)_2_SO_4_ (480.00 mg/L), NaCl (200.00 mg/L), MgSO_4_ · 7H_2_O (100.00 mg/L), and Na_2_CO_3_ (4000.00 mg/L). Store this solution in a refrigerator at 4 °C.

Solution B: During preparation, continuously introduce high-purity CO_2_ (99.99%) for 18 h until the solution attains a light pink hue. The components consist of EDTA (500.00 mg/L), FeSO_4_ · 7H_2_O (200.00 mg/L), MnCl_2_ · 4H_2_O (200.00 mg/L), ZnSO_4_ · 7H_2_O (10.00 mg/L), H_3_BO_3_ (30.00 mg/L), CoCl_2_ · 6H_2_O (20.00 mg/L), CuCl_2_ · 2H_2_O (1.00 mg/L), NiCl_2_ · 6H_2_O (2.00 mg/L), and NaMoO_4_ (3.00 mg/L). Store this solution in a refrigerator at 4 °C.

Solution C: Weigh 25 g of Na_2_S · 9H_2_O, dissolve it, and dilute it to a final volume of 100 mL. Introduce CO_2_ for 20 min and store at 4 °C in a refrigerator (prepare and use immediately).

On the day of the experiment, mix Solutions A, B, and C in a volume ratio of 988:10:2, and continuously introduce CO_2_ until the solution becomes transparent to prepare the fermentation buffer. Preheat the buffer to 39 °C for subsequent use.

#### 5.3.3. Preparation of Toxin Solution

ZEN-14G of the standard was accurately weighed and dissolved in chromatographic-grade methanol to prepare a single standard stock solution at a concentration of 1 mg/mL. This solution was stored at 4 °C and protected from light, and remained viable for one week. The total volume of the working solution for each treatment group was 300 μL; specifically, the CON group received 300 μL of methanol. In the GL group, 30 μL of the ZEN-14G mother liquor was added, and the volume was adjusted to 300 μL with methanol. The GH group received 300 μL of the ZEN-14G stock solution.

### 5.4. Rumen Fluid Collection and Culture

Rumen contents were collected from five healthy donor cattle via rumen fistulas prior to morning feeding (7:00 a.m.). These contents were mixed in equal proportions to obtain a representative mixture that minimized inter-individual variation [28], and were then placed in a thermos flask preheated to 39 °C and filled with CO_2_. After filtering through four layers of gauze to remove feed residue and large microbial aggregates, the mixture was combined with preheated fermentation buffer at a volume ratio of 1:2 (40 mL of gastric juice to 80 mL of buffer) to create the fermentation liquid. This liquid was then transferred into 150 mL vials, which were continuously purged with CO_2_ to maintain anaerobic conditions. Each vial received 2 g of the basic diet and 30 μL of the corresponding treatment group working solution, and the vials were incubated in a constant-temperature water bath shaker at 39 °C, operating at a speed of 80 r/min, for cultivation.

### 5.5. Sample Collection and Preservation

At 6 h, 12 h, and 24 h of fermentation, we removed the corresponding flasks and terminated fermentation by placing them in an ice bath. We immediately measured the pH of the fermentation broth using a pH meter. The remaining fermentation broth was aliquoted into 1.5 mL centrifuge tubes (1 mL per tube), rapidly frozen with dry ice, and transferred to a −80 °C freezer for storage. These samples were reserved for subsequent analysis of ammonia nitrogen, short-chain fatty acids, toxin metabolites, microbial communities, and metabolomics.

### 5.6. Indicator Measurement

#### 5.6.1. Fermentation Parameter Measurement

pH value: This was measured directly by inserting a portable pH meter (MP523-04, Shanghai Sanxin Instruments, Shanghai, China) into the fermentation broth. Each sample was tested three times, and the average value was recorded.

Ammonia Nitrogen (NH_3_-N): For this method, we employed the indophenol blue colorimetric method. We centrifuged 1 mL of fermentation broth at 4 °C and 10,000× *g* for 10 min. We then took 0.1 mL of supernatant and followed the instructions of the Ammonia Nitrogen Assay Kit (Nanjing Jiancheng Bio, Nanjing, China). We measured the absorbance at 570 nm using the UV-Vis spectrophotometer (UV-1201, Shimadzu, Kyoto, Japan) and calculated the content based on the standard curve.

Nutrient Loss Rate: After fermentation, we centrifuged the fermentation broth at 4 °C and 10,000× *g* for 15 min. We collected the precipitate, dried it at 65 °C to a constant weight, and determined the residual dry matter (DM) content; crude protein (CP) was determined by the Kjeldahl method, crude fat (EE) by the Soxhlet extraction method, and NDF and ADF by the Van Soest method. Loss rates were calculated using the formula:

Loss rate (%) = (Dietary nutrient content − Residual nutrient content)/Dietary nutrient content × 100.

#### 5.6.2. Targeted Analysis of Toxin Metabolites

Ultra-performance liquid chromatography–triple quadrupole mass spectrometry (UPLC-MS/MS) was employed to determine the concentrations of ZEN, ZEN-14G, and their metabolites (α-ZEL, β-ZEL, α-ZAL, β-ZAL, ZAN) in fermentation broth. Instrument model: Agilent 1290 Infinity II/6470 Triple Quadrupole LC/MS (Agilent Technologies, Santa Clara, CA, USA). Chromatographic conditions—column: ZORBAX SB-C18 (2.1 mm × 100 mm × 1.8 μm); mobile phases: A = 0.1% formic acid aqueous solution, B = acetonitrile; gradient elution: 0–2 min 30% B, 2–8 min 30–90% B, 8–10 min 90% B, 10–10.1 min 90–30% B, 10.1–15 min 30% B; flow rate: 0.3 mL/min; column temperature: 40 °C; injection volume: 5 μL. Mass spectrometry conditions—Electrospray ionization (ESI), positive-ion mode, multiple reaction monitoring (MRM); spray voltage: 3500 V; desiccant temperature: 350 °C; desiccant flow rate: 10 L/min; nebulizer pressure: 40 psi; MRM ion pairs and collision energies for each compound optimized based on a previously established method [29,30].

#### 5.6.3. Microbial Community Analysis

Samples of 24 h fermentation broth from the CON group and GH group (high-dose ZEN-14G group) were collected (eight replicates per group) and sent to Shanghai Meiji Biotechnology Co., Ltd., Shanghai, China for 16S rRNA gene sequencing. The procedure was as follows:

Total DNA extraction: Microbial total DNA was extracted using the E.Z.N.A.^®^ Soil DNA Kit (Omega Bio-tek, Norcross, GA, USA). Purity was assessed by a Nanodrop 2000 (Thermo Fisher Scientific, Waltham, MA, USA) (A260/A280 = 1.8–2.0), and integrity was verified by 1% agarose gel electrophoresis.

PCR Amplification: We targeted the V3-V4 region of the 16S rRNA gene using primers 341F (5′-CTACGGGNGGCWGCAG-3′) and 806R (5′-GACTACHVGGGTWTCTAAT-3′). Reaction mixture (25 μL): 5 × FastPfu Buffer 5 μL, dNTPs (2.5 mM) 2 μL, forward/reverse primers (10 μM) 1 μL each, FastPfu Polymerase 0.5 μL, DNA template 1 μL, ddH_2_O 14.5 μL. Reaction parameters: 95 °C pre-denaturation for 3 min, 95 °C denaturation for 30 s, 55 °C annealing for 30 s, 72 °C extension for 45 s (30 cycles), 72 °C final extension for 10 min.

Sequencing and Analysis: PCR products were purified via 2% agarose gel electrophoresis and sequenced using the Illumina NovaSeq PE300 platform (Illumina, Inc., San Diego, CA, USA). Raw data underwent quality control (removal of low-quality reads and adapter sequences), followed by denoising and inferring into amplicon sequence variants (ASVs) using the DADA2 pipeline. Species annotation was performed against the Silva 138 database. Alpha diversity (Ace, Chao, Shannon, Simpson indices), beta diversity (NMDS analysis), and differential species analysis (Kruskal–Wallis H test) were performed using the Meiji Cloud platform.

#### 5.6.4. Non-Targeted Metabolomics Analysis

Non-targeted metabolomics analysis was performed using the same samples as for microbial sequencing.

Sample Preparation: A 100 μL aliquot of fermentation broth was mixed with 1.5 mL of pre-chilled methanol/acetonitrile solvent (1:1, *v*/*v*), vortexed for 30 s, sonicated in a 4 °C water bath for 10 min, and stored overnight at −80 °C. After thawing, the mixture was centrifuged at 4 °C and 12,000 rpm for 15 min. The supernatant was collected, vacuum-dried (CV200, Beijing Jiaimu Technology Co., Ltd., Beijing, China), and reconstituted in 100 μL of methanol/acetonitrile (1:1, *v*/*v*). The solution was sonicated at 4 °C for 10 min, centrifuged at 12,000 rpm for 15 min, and stored overnight at −80 °C. Finally, the supernatant was transferred to autosampler vials.

Analysis was performed using an Agilent 1290 HPLC system coupled with an Agilent 6545 Q-TOF LC/MS quadrupole time-of-flight mass spectrometer (Agilent Technologies, Santa Clara, CA, USA): Eclipse Plus C18 column (RRHD, 1.8 μm, 2.1 × 100 mm) (Agilent Technologies, Santa Clara, CA, USA). Mobile phase A was 0.1% formic acid in water, and B was 0.1% formic acid in acetonitrile. Gradient elution was optimized based on metabolite characteristics. Mass spectrometry employed an ESI ion source with simultaneous detection in positive- and negative-ion modes, scanning *m*/*z* 50–1000.

Data Analysis: Raw data were processed using Progenesis QI software (peak identification, alignment, quantification) (version 3.0), then imported into SIMCA-P 14.1 for PCA (Principal Component Analysis) and PLS-DA (Partial Least Squares Discriminant Analysis). Criteria for selecting differential metabolites: VIP > 1 and nonparametric test *p* < 0.05 between groups. KEGG pathway enrichment analysis was performed using the MetaboAnalyst online platform.

#### 5.6.5. Establishment of a PCDL Database

A customized database was developed utilizing Agilent’s Personal Compound Database and Spectrum Library (PCDL) in conjunction with the Agilent 6545 Q-TOF LC/MS system. In reference to the 27 specific metabolites of ZEN-14G identified by Yang et al. [17], structural files of these metabolites in .mol format, along with their corresponding compound names and molecular formulas, were imported into the PCDL Manager B.08.00 software. The Profinder 10.0 software facilitated the extraction of batch target features from the sample data. By matching these features with the constructed PCDL database, rapid qualitative identification of target compounds within the samples was accomplished.

#### 5.6.6. Statistical Analysis

Sample sizes were appropriately allocated to meet the testing requirements for different biomarkers and omics approaches; these sample sizes were specifically designed based on various statistical requirements and analytical precision: each treatment was performed in eight independent in vitro fermentation replicates (vessels, *n* = 8). For comprehensive mechanistic exploration, all eight fermentation replicates from the 24 h incubation (CON and GH) were subjected to 16S rRNA gene sequencing and untargeted metabolomics. For targeted absolute quantification of ZEN-14G and its metabolites, five randomly selected fermentation replicates (*n* = 5) were analyzed via LC-MS/MS.

All statistical analyses were performed using R software (v4.2.1). Rumen fermentation characteristics (pH, NH3-N, VFAs, and nutrient digestibility) were analyzed using two-way ANOVA with Group, Time, and their interaction as fixed effects. Post hoc multiple comparisons between treatment groups at each time point were performed using Fisher’s LSD test, with statistical significance declared at *p* < 0.05. The results are presented as Mean ± SEM (standard error of the mean).

For 16S rRNA gene sequencing data (conducted at 24 h), differences in alpha diversity indices (including Ace, Chao1, Shannon, and Simpson) and the relative abundance of microbial taxa between the CON and GH groups were analyzed using the two-tailed independent Student’s *t*-test (for normally distributed data) or Wilcoxon rank-sum test (for non-normally distributed data). For beta diversity, differences in microbial community structures were visualized using Principal Coordinate Analysis (PCoA) and Principal Component Analysis (PCA), and significance was assessed by Permutational Multivariate Analysis of Variance (PERMANOVA, adonis2 function in the vegan package) based on Bray–Curtis distance matrices with 999 permutations.

For untargeted metabolomics, Principal Coordinate Analysis (PCoA) and Orthogonal Partial Least Squares Discriminant Analysis (OPLS-DA) were performed. Model validation of OPLS-DA was assessed by permutation tests (200 times). Differentially abundant metabolites (DAMs) were screened based on the Variable Importance in Projection (VIP > 1.0) and *p*-values (*p* < 0.05) from Welch’s *t*-test. Metabolic pathway enrichment and topology analyses were conducted using the MetaboAnalyst database.

Statistical graphics were generated using the ggplot2 package in R. Correlation analyses between differentially abundant microbial taxa and metabolites were performed using Spearman rank correlation, with significance levels denoted as * *p* < 0.05, ** *p* < 0.01, and *** *p* < 0.001.

## Data Availability

The original contributions presented in this study are included in the article/Supplementary Material. Further inquiries can be directed to the corresponding author.

## Supplementary Materials

The following supporting information can be downloaded at: https://www.mdpi.com/article/10.3390/toxins18090384/s1, Figure S1: Dose- and time-dependent effects of ZEN-14G on rumen fiber digestibility; Figure S2: Rumen microbial community alpha and beta diversity and Venn distribution under ZEN-14G treatment; Figure S3: Rumen microbial composition stacked bar charts at the phylum and genus levels; Figure S4: Rumen metabolome PCoA score plots.
