# Supplementary material for: Integrated Fermentation, Microbiome and Metabolomic Profiles Reveal Rumen Lipotoxicity Induced by the Masked Mycotoxin Zearalenone-14-Glucoside
Source: Toxins (Basel). 2026 Sep 6;18(9):384. doi: 10.3390/toxins18090384 (PMC13611239; doi:10.3390/toxins18090384)
Supplement: Supplementary file 1 [file toxins-18-00384-s001.zip › toxins-4478620-supplementary.pdf]

Supplementary Materials: Integrated Fermentation, Microbiome and Metabolomic Profiles Reveal Rumen Lipotoxicity Induced by the Masked Mycotoxin Zearalenone-14-Glucoside

Zixin Wang, Mingzhu Chen, Jialei Liu, Tao Wang, Zhe Sun and Xiaolu Jin

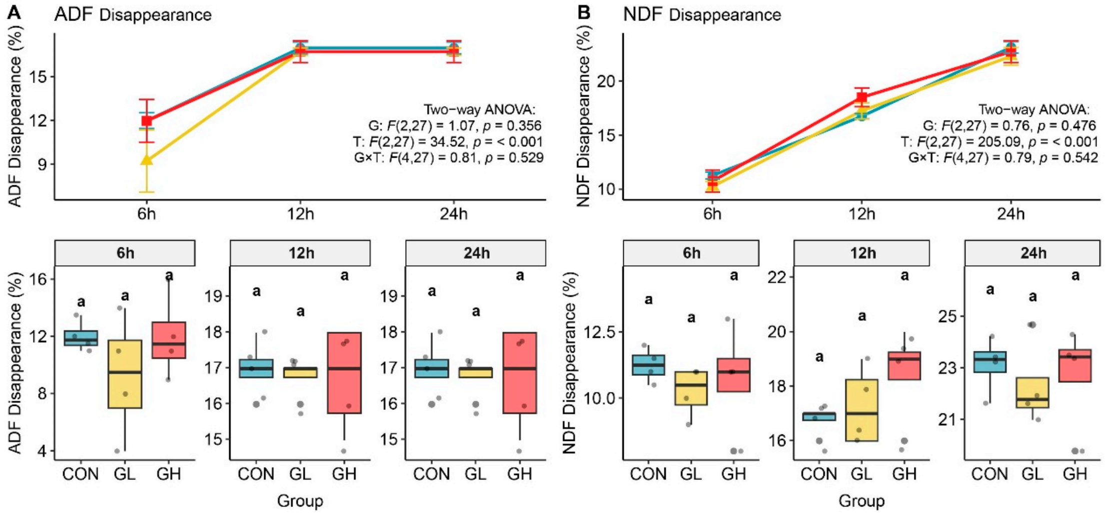

Figure S1. Dose- and time-dependent effects of ZEN-14G on rumen fiber digestibility. Panels represent (**A**) acid detergent fiber (ADF) disappearance and (**B**) neutral detergent fiber (NDF) disappearance. Each parameter is presented as a composite plot (trend line on top representing temporal changes, and boxplots on the bottom representing group distribution at 6, 12, and 24 h). Different lowercase letters (a, b, c) indicate significant differences (Fisher's LSD, $p < 0.05$).

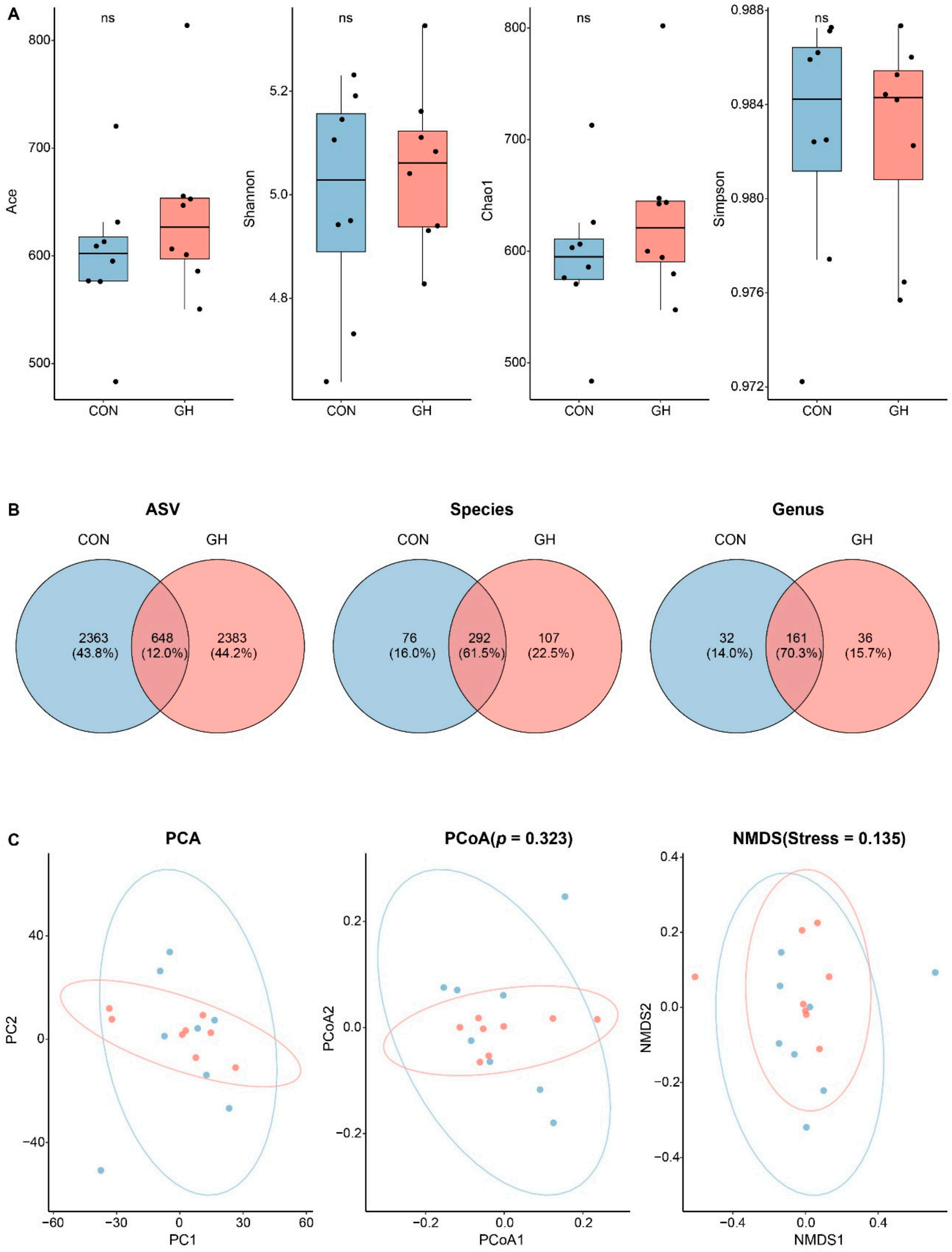

Figure S2. Rumen microbial community alpha and beta diversity and Venn distribution under ZEN-14G treatment. (**A**) Alpha diversity indices. Boxplots show the Ace, Shannon, Chao1, and Simpson indices for Control (CON, blue) and High-dose (GH, red) groups. Data are presented as Mean ± SEM. Statistical significance was analyzed by t-test. (**B**) Venn diagrams. Plots display the number of shared and unique features at ASV, Species, and Genus levels. (**C**) Beta diversity analysis. Scatter plots visualize the clustering of microbial communities using PCA, PCoA (based on Bray-Curtis distance, with PERMANOVA *p*-value), and NMDS (with Stress value). Ellipses indicate 95% confidence intervals.

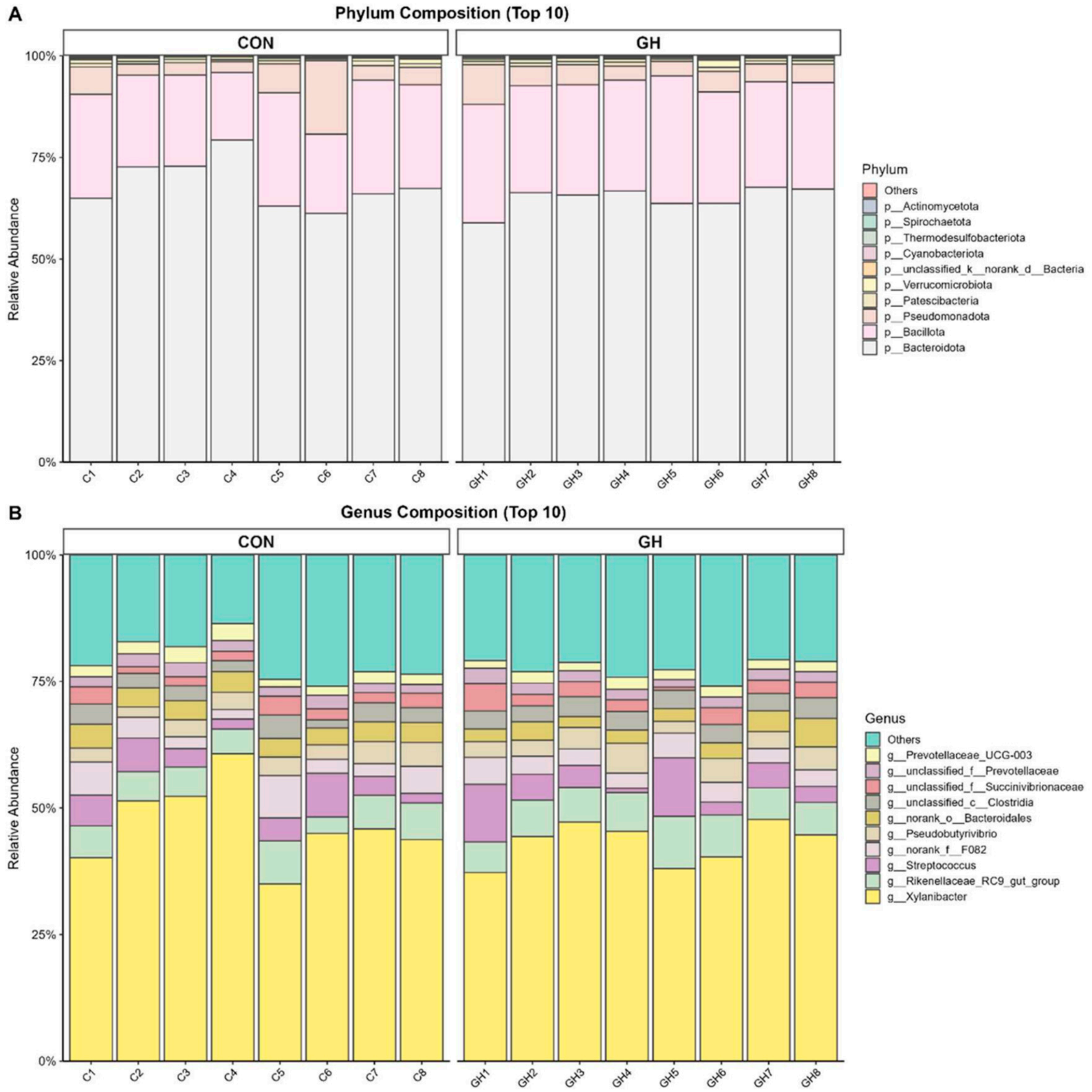

Figure S3. Rumen microbial composition stacked bar charts at the phylum and genus levels. (**A**) Phylum-level composition. Stacked bar chart displaying the relative abundance of the top dominant phyla for each individual sample. (**B**) Genus-level composition. Stacked bar chart displaying the relative abundance of the top dominant genera for each individual sample.

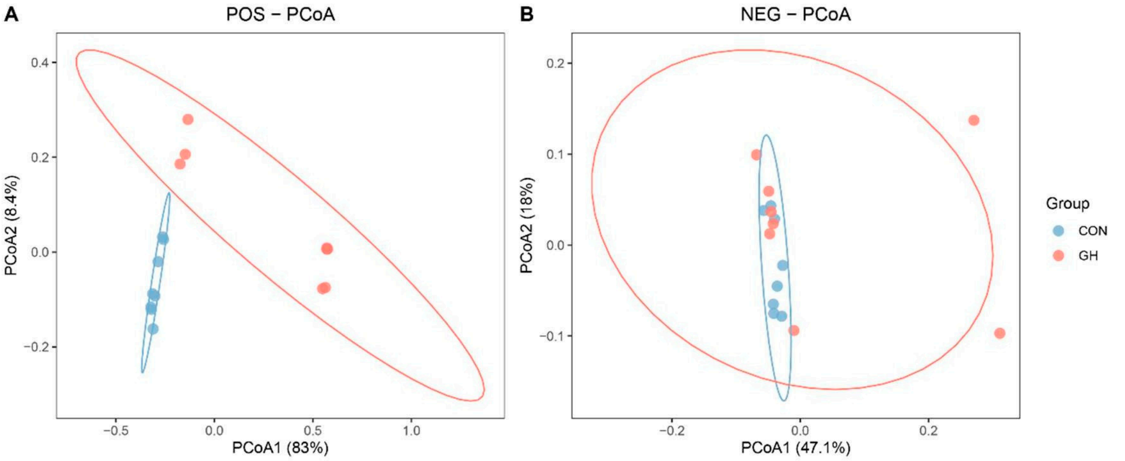

Figure S4. Rumen metabolome PCoA score plots. Principal Coordinate Analysis (PCoA, based on Bray-Curtis distance) score plots of (**A**) Positive Ion Mode (POS) and (**B**) Negative Ion Mode (NEG). Percentages indicate the proportion of variance explained by each coordinate. Ellipses indicate 95% confidence intervals.
